# Motor neuronopathy with dropped hands and downbeat nystagmus: A distinctive disorder? A case report

**DOI:** 10.1186/1471-2377-6-3

**Published:** 2006-01-12

**Authors:** Nimish J Thakore, Erik P Pioro, Janet C Rucker, R John Leigh

**Affiliations:** 1 Department of Neurology, MetroHealth Medical Center, 2500 MetroHealth Drive, Cleveland, OH 44109, USA; 2 Department of Neurology, Cleveland Clinic Foundation, 9500 Euclid Avenue, Cleveland, OH 44195, USA; 3 Department of Neurology, Rush University, 1725 West Harrison, Chicago IL 60612, USA; 4 Department of Neurology, Veterans Affairs Medical Center, 10701 East Boulevard, Cleveland, OH 44106, USA; 5 Department of Neurology, University Hospitals, 11100 Euclid Avenue, Cleveland, OH 44106, USA; 6 Department of Neurology, Case Western Reserve University, 11100 Euclid Avenue, Cleveland, OH, 44106, USA

## Abstract

**Background:**

Eye movements are clinically normal in most patients with motor neuron disorders until late in the disease course. Rare patients are reported to show slow vertical saccades, impaired smooth pursuit, and gaze-evoked nystagmus. We report clinical and oculomotor findings in three patients with motor neuronopathy and downbeat nystagmus, a classic sign of vestibulocerebellar disease.

**Case presentation:**

All patients had clinical and electrodiagnostic features of anterior horn cell disease. Involvement of finger and wrist extensors predominated, causing finger and wrist drop. Bulbar or respiratory dysfunction did not occur. All three had clinically evident downbeat nystagmus worse on lateral and downgaze, confirmed on eye movement recordings using the magnetic search coil technique in two patients. Additional oculomotor findings included alternating skew deviation and intermittent horizontal saccadic oscillations, in one patient each. One patient had mild cerebellar atrophy, while the other two had no cerebellar or brainstem abnormality on neuroimaging. The disorder is slowly progressive, with survival up to 30 years from the time of onset.

**Conclusion:**

The combination of motor neuronopathy, characterized by early and prominent wrist and finger extensor weakness, and downbeat nystagmus with or without other cerebellar eye movement abnormalities may represent a novel motor neuron syndrome.

## Background

Eye movements are usually spared in motor neuron disorders, such as amyotrophic lateral sclerosis (ALS), spinal muscular atrophy, Kennedy's disease and poliomyelitis. In ALS, the eye movement examination typically remains normal until late in the course of the disease, despite severe weakness of the skeletal and bulbar muscles[1]. Neuropathological studies have indicated that ocular motoneurons are spared except in patients with advanced disease who had been maintained on respirators prior to death[2,3]. Laboratory testing has demonstrated defects of memory-guided saccades and increased errors on the antisaccade task, consistent with frontal lobe involvement in ALS[4,5], but such defects are not usually recognized at the bedside. Standing apart from this classic picture is a subset of patients ALS in whom disordered eye movements are clinically evident early in the course. Such patients are variously reported to show slowing of vertical saccades [6], gaze-evoked horizontal or rotary nystagmus[7], and impairment of smooth pursuit[8]. Ocular motor abnormalities may be more common in cases with dementia[2], prominent bulbar involvement [9], or features of parkinsonism[8].

Apart from ALS, some specific disorders may have prominent oculomotor abnormalities associated with motor neuron degeneration. These include late-onset Tay-Sachs disease (hexosaminidase A deficiency)[10], the Guamian syndrome[11], spinocerebellar ataxia type 3 (SCA3)[12], and multiple system atrophy[13].

We report 3 patients with a distinctive, sporadic motor neuronopathy characterized by early wrist drop, prolonged survival, and downbeat nystagmus. The first two were middle-aged women with progressive muscle weakness who developed visual complaints. The third was a man fortuitously identified during chart review for a study of posterior interosseous neuropathy. In the first two patients, we recorded eye movements using the magnetic search coil technique, measuring fixation in central and eccentric gaze angles, saccades, pursuit, the vestibulo-ocular reflex (VOR) and vergence, as previously described [10]. Both patients gave informed, written consent of the protocol, which was approved by the Institutional Review Board of the Cleveland Veterans Affairs Medical Center.

## Case presentation

**Patient 1 **was 47 years old when we assessed her. Onset was at age 16 years with cramping and twitching of extremity muscles and finger extension weakness. Weakness worsened during pregnancy at the age 21 years, and also involved the knees. EMG revealed widespread fibrillation and chronic neurogenic motor unit potential changes. Left deltoid muscle biopsy showed changes of chronic denervation. A diagnosis of late-onset spinal muscular atrophy was made at the age of 22 years. About that time she first noticed diplopia on lateral gaze to either side, with diagonal separation of the images. Over the ensuing 25 years, her leg weakness progressed to the point that she needed a cane, and often used a wheelchair. Finger and wrist extension weakness was severe, but she could still flex her fingers, grip her cane, and propel her wheelchair. She had also noted the development of vertical oscillopsia (illusory motion of the visual environment) when she viewed distant objects. She had no bulbar or respiratory symptoms. Additional medical history included Heller's myotomy performed for achalasia, gastroesophageal reflux symptoms and obesity. Family history was remarkable only for poliomyelitis in her mother.

On examination, visual acuity was 20/20 in each eye. Visual fields and optic fundi were normal. Her range of eye movements was full. There was low-amplitude downbeat nystagmus, evident in central position during ophthalmoscopy, which increased in lateral and down gaze. Saccades were normal. Pupils were 3 mm and reactive. Muscles of the face and tongue were strong, without evident fasciculations. There was no neck, pharyngeal or respiratory weakness. In the arms, there was diffuse rather symmetrical mild weakness (MRC grades 4 to 4 plus) of most muscles, and severe weakness of wrist and finger extensors (grade 2), causing bilateral finger and wrist drop. The legs were also diffusely weak, grades 4 minus to 4. Muscle bulk and fasciculations were difficult to assess because of obesity. Muscle tone was normal. There was no dysmetria, and rapid alternating movements were normal. Deep tendon reflexes were normal and symmetrical in the upper extremities, hypoactive at the knees, and absent at the ankles. Plantar responses were flexor. Sensory examination was normal. The patient managed to walk rather slowly with a cane. MRI of the brain was unremarkable with the exception of scattered non-specific T2-hyperintensities in periventricular white matter. Neither cerebellar atrophy nor tonsillar ectopia was seen. Vitamin B12, folate, vitamin E, TSH, and lead levels in blood were normal. Genetic tests for spinocerebellar ataxia (SCA) mutations, SCA3 and SCA6, and the common spinal muscular atrophy mutation (SMN1) were negative. Hexosaminidase A deficiency was not found. Anti-nuclear antibodies were detected in low titer (1:40, speckled pattern), but double-stranded DNA and extractable nuclear antibodies were absent. Anti-gliadin antibody (IgA-type) was elevated in serum (42.1 EU, normal 0–29.9, ARUP Laboratories, Salt Lake City, Utah), but anti-gliadin antibody (IgG-type) and anti-transglutaminase antibodies were normal or undetectable. Anti-glutamic acid decarboxylase (GAD) antibody was elevated in serum (GAD65 17.4 U/ml, normal <1.5 U/ml, Specialty Laboratories Inc, Valencia, California). CSF was normal and revealed neither detectable anti-GAD antibody nor evidence of intrathecal antibody synthesis.

**Figure 1:**
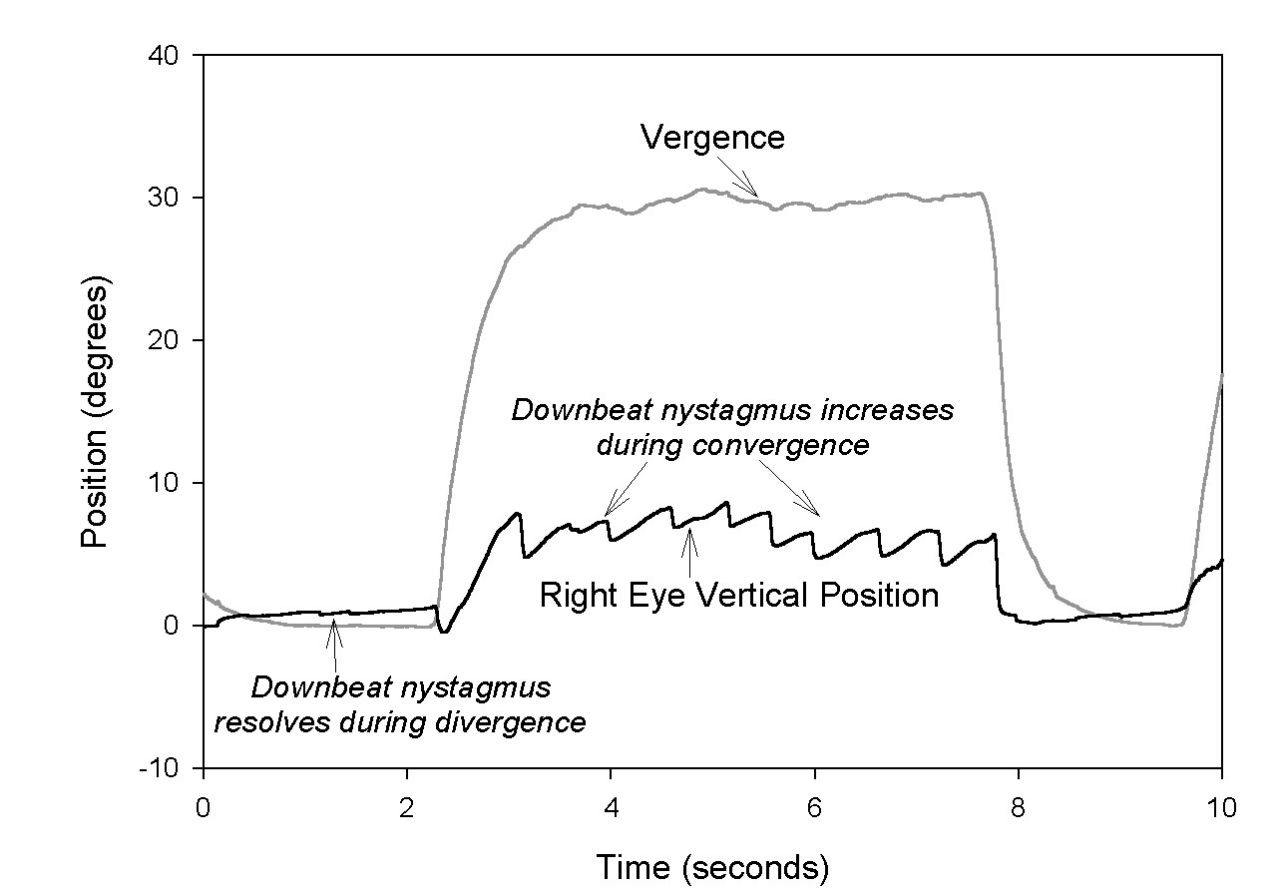
Representative record of downbeat nystagmus in Patient 1, which increased with convergence. Positive values indicated rightward, upward, or convergence movements.

Eye movement measurements confirmed downbeat nystagmus, which increased on lateral and down gaze, following head shaking (especially vertically) and during convergence (Fig. 1). She had an alternating skew deviation, with hypertropia of about 1.5 degrees in the abducting eye. Saccades were accurate and of normal velocity; there was no internuclear ophthalmoplegia. The VOR was normal in both the horizontal and vertical planes.

**Patient 2 **was 46 years old when we assessed her. She had first noted balance and gait problems with occasional falls at 38 years age. This was associated with subjective vertigo, intermittent blurring of vision, vertical diplopia on lateral gaze, and oscillopsia with head turning to the left. At about the same time, there was progressive weakness of finger extension of her right hand, "one finger at a time", accompanied by hand and arm cramping. Shortly thereafter, the left hand was similarly involved. Finger flexors remained relatively strong. More recently, mild weakness in the arms and shoulders, and difficulty getting out of a car or standing for prolonged periods had developed, and fasciculations had increased in the legs. She had no bulbar or respiratory symptoms. Her past health had been good otherwise with no family history of similar problems.

On examination, visual acuity with correction was 20/25 in each eye. Optic fundi and visual fields were normal. She had a full range of eye movements, but downbeat nystagmus was evident, especially on lateral and down gaze. Saccades, pursuit, vergence and VOR were clinically normal, but intermittent horizontal ocular oscillations were noted. The remaining cranial nerves were normal. Muscle atrophy was limited to intrinsic hand muscles. Tone was diminished in the upper extremities, but slightly increased in the legs. Intrinsic muscles and finger extensors were profoundly weak, especially on the right (grade 1). Wrist and elbow extensors were moderately weak, slightly worse on the right (grade 4), while finger, wrist, and elbow flexors were only minimally affected. There was no appreciable weakness of lower extremities, although gait was slightly slow and stiff. Deep tendon reflexes were diminished in the arms but pathologically brisk in the legs with horizontal spread. Bedside spirometry revealed normal forced vital capacity.

**Figure 2:**
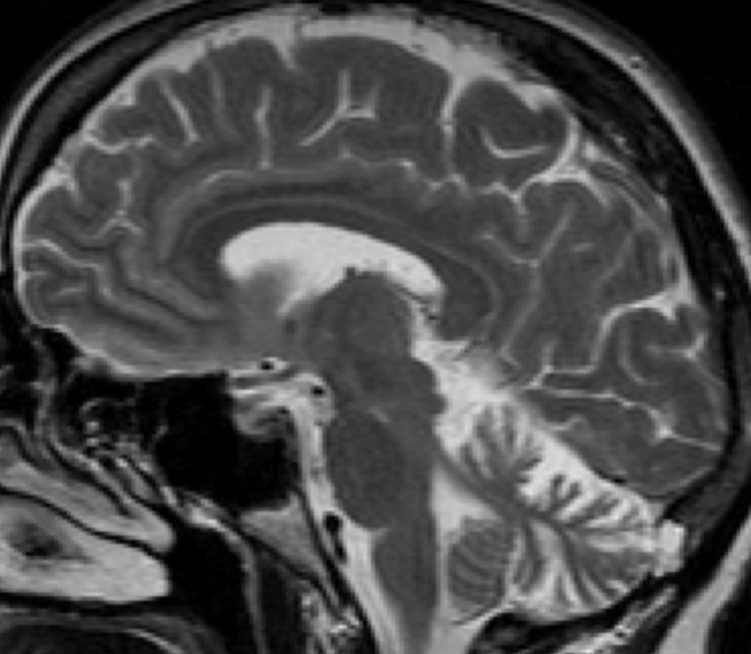
MRI brain of patient 2 showing atrophy of cerebellum.

EMG done 3 years after onset showed evidence of slowly progressive motor neuron degeneration with mild fibrillations in a patchy distribution throughout the upper extremity and thoracic paraspinal muscles. Changes of chronic motor axon loss were noted in most muscles of the upper extremity and in some muscles of the lower extremity. Motor conduction blocks were not observed. Normal or negative laboratory tests included blood and 24-hour urine lead levels, HIV, blood hexosaminidase A, ceruloplasmin, vitamin E, and amino acid levels, genetic tests for spinocerebellar ataxia (SCA) types 1,2,3,6,7 and 8 and Friedreich's ataxia, and urinary organic and amino acids. Vitamin B12 level was borderline (214 pg/mL, normal > 221), while folate level was normal. Anti-nuclear nuclear antibodies were present (1:640, homogenous pattern), with elevated anti-DNA antibody. Extractable nuclear antibodies were absent. Anti-GAD, anti-gliadin, anti-endomysial and anti-transglutaminase antibodies and paraneoplastic autoantibodies (anti-Hu, -Ro and Ri) were not detected in serum. CSF was normal without evidence of intrathecal antibody synthesis. MRI of the brain and cervical spinal cord revealed mild atrophy of the cerebellum, particularly the superior aspects of the vermis (Fig. 2), and mild atrophy of the upper cervical cord. Proton magnetic resonance spectroscopy demonstrated diminished *N*-acetylaspartate signal in the posterior limb of the left internal capsule, suggesting corticospinal axon involvement. Gastrocnemius biopsy revealed chronic active neurogenic atrophy, while sural nerve biopsy revealed mild loss of myelinated axons with demyelinative changes. Analysis of fresh muscle for acetylcarnitine and electron transport chain abnormalities was unrevealing.

**Figure 3:**
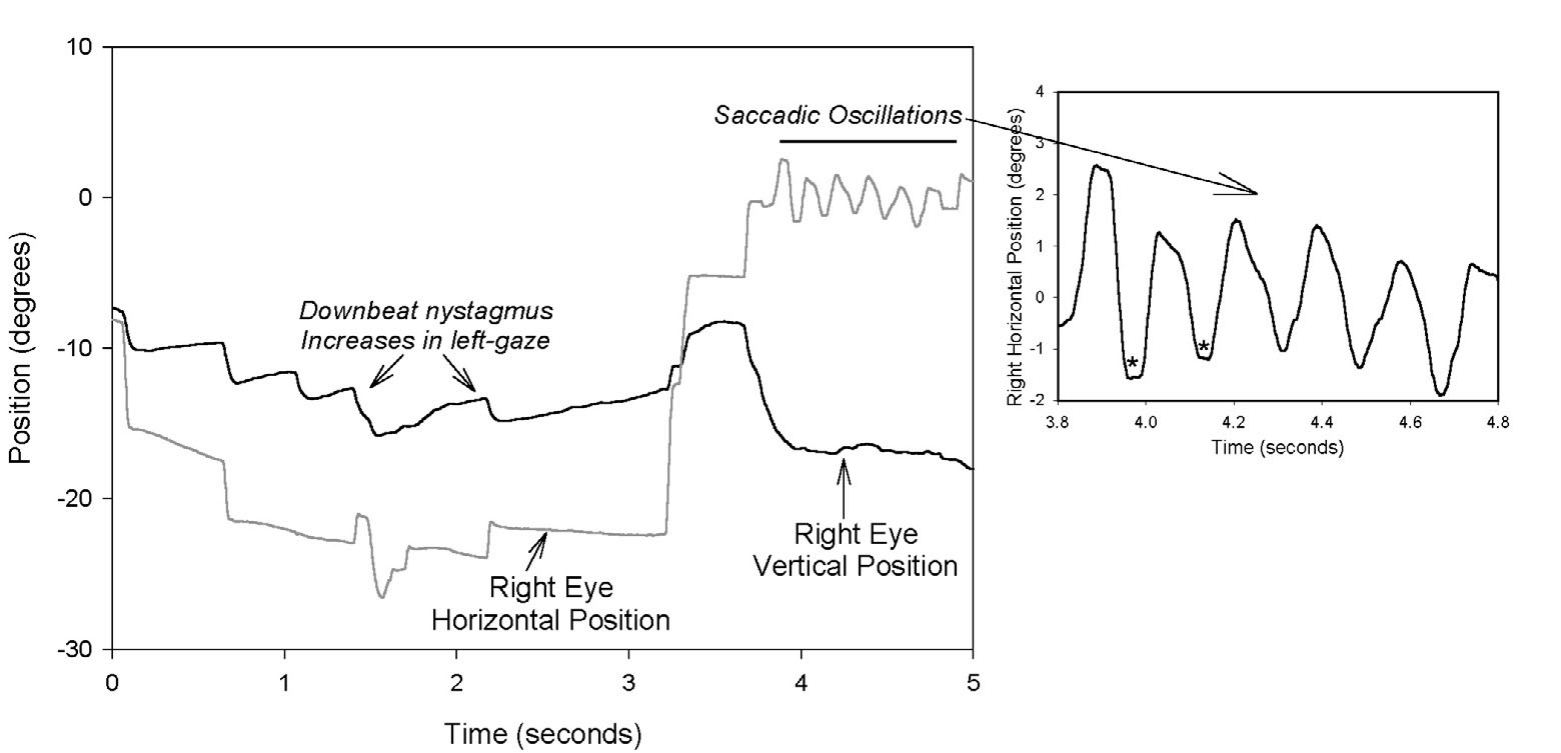
Representative record from Patient 2, showing downbeat nystagmus on left-gaze and a burst of horizontal saccadic oscillations. On the inset at right, the saccadic oscillations are shown at a different time scale; note that an intersaccadic interval (marked by asterisk) is present for some, but not all, cycles. Same conventions as in Figure 1.

Eye movement measurements confirmed that she had downbeat nystagmus on lateral and downgaze (Fig. 3), but this did not increase with convergence, or after head shaking. In addition, she showed intermittent horizontal saccadic oscillations, occurring in bursts, at a frequency of about 5–6 Hz; brief intersaccadic intervals could be discerned for some, but not all, oscillation cycles (subplot in Fig. 3). Otherwise, horizontal and vertical saccades were accurate and of normal velocity; there was no internuclear ophthalmoplegia. Smooth pursuit was impaired both horizontally and vertically. The VOR was normal in both horizontal and vertical directions. However, during horizontal head rotations, movements to the left induced an upward movement, and subsequent corrective saccade.

**Patient 3 **was examined by various neurologists at two institutions between 1984 and 1990 and was subsequently lost to follow-up. His first symptoms were weakness of extension of the left little finger in 1982 at the age of 40 years. This had spread to digits 3 and 4 of the left hand, but was not accompanied by sensory change or pain. His left radial nerve was decompressed in 1984 without benefit. By this time, milder weakness of finger and wrist extensors was also noted on the right. EMG in 1984 and 1985 revealed evolving changes of active and chronic denervation spreading from the posterior interosseous distribution to the radial distribution bilaterally, with sparing of sensory responses. Motor conduction block was not observed. The right radial nerve was decompression in 1986, again without benefit. Between 1986 and 1990, the disorder progressed to a clinically obvious anterior horn cell disorder predominantly affecting the arms, with wrist drop and finger drop, gross shoulder girdle, arm and forearm atrophy with fasciculations, chest fasciculations, and mostly preserved leg and cranial musculature. Deep tendon reflexes were lost in the arms, normal at the knees and hypoactive at the ankles. Sensory examination was normal. EMG in 1990 revealed widespread active and chronic motor axon loss with fasciculations in the arms, and subtle neurogenic changes in thoracic and lumbosacral myotomes. Identification of downbeat nystagmus prompted referral to a neurologist with expertise in eye movements (Dr. Robert B. Daroff) in 1990, who confirmed the finding, noted that it was accentuated by lateral gaze and reduced by upward gaze, and observed that it had been symptomatic since 1989 with vertical oscillopsia. MRI of the brain was unremarkable except for non-specific periventricular white matter changes, and revealed neither cerebellar atrophy nor tonsillar ectopia. There was a history of exposure to trichloroethylene and lead batteries, but tests for heavy metals were negative. Blood and urine tests revealed slight elevation of cryoglobulins and mildly positive antinuclear antibodies (1:40 speckled). Numerous other laboratory tests, including serum and urine immunoelectrophoresis and tests for hexosaminidase A, Kennedy's disease, GM1 antibodies, and porphyria were negative. There was no family history of similar neurological disease.

## Conclusion

The key features of the syndrome we have described are (1) clinical and electrodiagnostic findings of progressive loss of anterior horn cells, (2) early and disproportionately severe involvement of finger and wrist extensors, (3) relative sparing of facial, pharyngeal, tongue and respiratory musculature, (4) prolonged survival, (5) lack of a family history of similar disorder, and (6) eye movement abnormalities, particularly downbeat nystagmus. Variable features include some degree of upper motor neuron involvement (Patient 2), cerebellar atrophy on imaging (Patient 2), and significant lower extremity weakness (Patient 1). To our knowledge, a similar disorder has not been previously described. Coexistence of motor neuronopathy and oculomotor disorder could well be coincidental, such as from the chance overlap of cerebellar degeneration and motor neuron disease in patient 2. The association of an unusual nystagmus and with a specific topographical distribution of weakness in 3 patients from one geographical region in the United States, however, suggests otherwise.

The neuroanatomical basis of oculomotor involvement in these cases in uncertain, but some features point to the cerebellum. Several hypotheses have been proposed to account for the pathogenesis of downbeat nystagmus[1,14]. These include asymmetric inhibition of the vertical VOR, imbalance of otolithic inputs, dysfunction of the mechanism that holds gaze steady (the neural integrator for eye movements) or a disorder of vertical smooth pursuit. In each of these hypotheses, a cerebellar role in the pathogenesis of downbeat nystagmus is thought to be most likely [14]. Impaired smooth pursuit and gaze-evoked nystagmus, which have previously been noted in ALS[7,8], may be signs of cerebellar disease[1] Alternating skew deviation (patient 1) has also been attributed to cerebellar disease[1]. Saccadic oscillations are less well localized[1]. Patient 2 showed 6-Hz conjugate horizontal oscillations that sometimes showed brief intersaccadic intervals, but at other times appeared to be back-to-back flutter movements (Fig. 3). Although the pathogenesis of ocular flutter is debated[1], similar oscillations have been described in patients in whom fMRI showed increased activity in the cerebellar fastigial nuclei[15]. MRI in Patient 2 did reveal cerebellar atrophy, but this finding was not observed in the other two cases. Of note, none of the patients had classical signs of cerebellar involvement, such as dysmetria, dysdiadochokinesis or dysarthria. Although there was clear evidence of motor neuronopathy with electrophysiological sensory preservation in all cases, areflexia (Patient 1) and subtle sural nerve biopsy abnormalities (Patient 2) may suggest mild sensory fiber involvement.

What could cause this disorder? Although a degenerative process appears likely, the disorder is clearly not ALS as we know and define it[16]. Testing in some patients has excluded a few of the heredofamilial disorders that might have a similar phenotype, namely SCA3, SCA 6 and hexosaminidase A deficiency. Lead toxicity was excluded in all patients. Anti-nuclear antibodies were detected in all cases, but none had systemic symptoms or signs suggestive of systemic lupus erythematosus or another defined connective tissue syndrome. Although we found modest elevation of anti-glutamic acid decarboxylase (GAD) and IgA anti-gliadin antibodies in the serum of one case (Patient 1), the role of these antibodies in the pathogenesis of this disorder is uncertain because of their absence intrathecally. Anti-GAD antibodies have been reported with idiopathic downbeat nystagmus[17], but the specificity of this association has been questioned because other disorders of eye movements have also been noted with these antibodies [18]. Likewise, anti-gliadin antibodies have been associated with sporadic ataxia, but are indeed nonspecific for their neurological associations, and quite prevalent in the general population[19,20]. We admit that the diagnostic evaluation of these patients, while extensive, is not complete, and may have missed an unusual but known cause of such as syndrome.

Although these patients were sufficiently unusual to warrant reporting, validation of the existence of motor neuronopathy with dropped hands and downbeat nystagmus as a distinctive disorder will have to await recognition of additional patients.

## Competing interests

The author(s) declare that they have no competing interests.

## Authors' contributions

NT conceived of the study, examined patients 1 and 2, identified patient 3, and drafted part of the manuscript. EP examined patient 2, drafted part of the manuscript. JR examined patients 1 and 2, recorded eye movements, and drafted part of the manuscript. RJL examined patients 1 and 2, recorded eye movements, and drafted part of the manuscript. All authors read and approved the final manuscript.

## Pre-publication history

The pre-publication history for this paper can be accessed here:
